# General Review of Medical Progress and News

**Published:** 1872-11

**Authors:** J. H. Etheridge

**Affiliations:** Chicago


### Chicago Medical Journal.

A MONTHLY RECORD OF

*Medicine, Surgery and the Collateral Sciences.*

EDITED BY J. ADAMS ALLEN, M.D., LL.D.; AND WALTER HAY, M.D.

---

VOL. XXIX. — NOVEMBER, 1872. — NO. 11.

---

### Original Communications.

ARTICLE I. — *General Review of Medical Progress and News.* Prepared by J. H. ETHERIDGE, M.D., Chicago.

### "PARTURITION NOT NECESSARILY A PAINFUL PROCESS."

Under the above caption, Dr. J. S. Wilson, of Atlanta, Ga., in the *Atlanta Medical and Surgical Journal*, for August, 1872, offers some very refreshing and sensible remarks on an old subject. He first reviews the nervous supply of the uterus, and in it finds no reason for expecting pain when the organ is in a normal condition. Great emphasis is laid upon this "normal condition," and as all physicians know that parturition is or ought to be a "normal" process, he certainly has much, theoretically, to confirm his assertions. Secondly, he substantiates his position practically or experimentally, and herein he becomes intensely interesting to every medical man. Hear him:

"Case 1. Mrs. ——, had been confined three times—each labor very severe—and has suffered from most of the multiform 'diseases of pregnancy.' In her fourth pregnancy she was subjected to the hygienic course (yet to be mentioned), and escaped most of the ailments of pregnancy, while parturition was effected in three hours from the beginning, with an almost painless expulsive stage of half an hour, the work being accomplished with only three or four contractions. In her fifth pregnancy she had the same or even greater immunity from the diseases of pregnancy than in the fourth, under similar treatment. In this she was delivered in three hours from the beginning of labor, with not more than two expulsive efforts, and these were attended with scarcely any pain. In her sixth labor, dilatation was accomplished in twelve hours by *painless contractions*, and expulsion was effected in twenty minutes, with six strong and almost painless contractions; and this after a non-child-bearing period of twelve years, during most of which time she had suffered more or less from ulceration of the womb."

"Case 2. Mrs. C., æt. 17—primapara—short stature, firm tissues; closely built but well formed. Subjected to preparatory treatment; was delivered in about six hours from the beginning of labor, with a very short and almost painless expulsive stage. So little complaint was made that I hardly knew when the child was born, though present all the time; and the patient did not suppress her complaints, for she expressed surprise that her labor was over so soon and with so little pain. There was no hemorrhage, no bad smell, or other disagreeable accompaniment of childbed."

"Case 3. Mrs. S., æt. 18—well formed—health good—primapara—subjected to preparatory regimen. Labor six hours—expulsive stage short, only one or two contractions finished it, and she said she felt scarcely any pain. In her second confinement, after the same previous treatment, she was sick three or four hours, with expulsive stage twenty minutes long and almost painless. No accidents—recovery good. In her next labor she was sick only forty-five minutes from first pain; only nine contractions in all, seven dilatatory and two expulsive, almost painless."

"Case 4. Mrs. F., æt. 25—good health—well formed—third labor. Previous two labors severe. Treated in this (the third) with usual preparation. Labor four or five hours—expulsive stage thirty minutes, little pain; no accidents of any kind."

"Case 5. Mrs. H., æt. 18—form and health good, but had been delicately raised. Treatment as in previous cases. Labor six hours—expulsive stage forty-five minutes—scarcely any pain; no accidents of any kind."

A short analysis of the cases reported is then given, and the writer passes on to consider the Bible aspect of labor. Every physician, with a heart in him, most assuredly agrees with Dr. W. in the expediency of lightening labor-sufferings as much as possible. A review of medical literature of the past twelve or fifteen years shows many acrimonious disputes as to the duties of physicians in using or not using chloroform in the lying-in room: one side claiming that it is wrong, because it was said to woman after the fall, "In sorrow [pain] shalt thou bring forth children."

The principal means of the preparatory treatment may be thus summed up: "Good, digestible, laxative diet, in *moderate* quantities; cooling, bland, unstimulating drinks; avoidance of stooping or doubled positions; loose, comfortable clothing; pure air by day and night; abundant sleep; sunlight; and, last though not least, general and local baths, followed by friction of the skin."

Thus it is seen that nought but hygienic measures are resorted to—no medicines or medication of any kind. Hygienists have long claimed that pregnant women ought to live as thus recommended, and have also claimed for them precisely the same results, so admirably set forth by Dr. Wilson. If our memory serves us rightly, Mrs. Elizabeth Cady Stanton, before a Chicago audience, two years ago, detailed the particulars of her personal experience in pregnancy with this preparatory treatment, with an almost painless labor following.

In particularizing, it may be necessary to quote the writer's words on bathing: "To subdue the nervous and vascular excitement incident to pregnancy, and to depurate the blood, there is nothing so safe and effectual as tepid, cool, or even cold baths. These baths abstract excessive heat, equalize the circulation, remove internal congestions, and strengthen the whole system, thus preparing it to furnish suitable elements for the new being, and to pass safely through the critical time of parturition. The feelings of the patient may be safely consulted as to the temperature of the water; the sponge bath or wash-down is the best mode of applying it. Of course, the shower bath, and all applications that cause violent shock, should be avoided. Pregnant women generally bear cold water remarkably well, but they cannot well tolerate violent impressions of any kind. The sponge bath should be used every day, or every other day, from the beginning of pregnancy, and the hip bath should be resorted to once a day during the last month or two. The temperature of this should be moderate, and it should not be continued more than five or ten minutes each time. The cold hip bath, with the general baths recommended, places the system in the most favorable condition for parturition, and, I may add, is one of the most, if not the most, effectual of all the means at our command for preventing or allaying that morbid irritability and that excessive rigidity which are the great, the principal, if not the sole, causes of the pains of labor."

Cannot some readers of the JOURNAL put these precepts in force, and, within the ensuing year, write out their experience for publication?

ELECTROLYSIS IN OVARIAN TUMORS.

Dr. Fieber reports, in the *Vienna Medical Press*, a cure of an ovarian tumor by means of electricity through needles, and earnestly recommends in all cases the trial of this means before facing the awful chances of ovariotomy. He says:

"A seamstress, æt. 32 years, had a tumor of the size of a man's head, slightly painful on pressure, hard, nodulated, extending from half an inch below the umbilicus down to within an inch above the symphysis; its breadth was six inches. Diagnosis: ovarian cyst of the right side.

"June 6th, 1870, a large gold needle with a copper pole, connecting with a Daniels battery of twenty elements, was inserted one and a half inches below and to the right of the umbilicus, the chain was then closed to the left of the umbilicus. While the needle was inserted, the patient experienced a sensation of severe burning, which, however, soon disappeared. After seven minutes the needle was withdrawn; reaction slight.

"Up to Nov. 30th, 1870, electrolysis was repeated eleven times. Subsequent inflammatory manifestations occurred, but rarely requiring poultices and small doses of morphine. The needle was inserted into different places. Till the end of November the swelling diminished but little; afterwards it decreased rapidly. March 10th, 1871, the cyst was reduced to the size of a hen's egg."

All experimenters in the application of electrolysis to tumors agree that at first, in most cases, the reduction in size of these growths proceeds with marked slowness for months, when suddenly they disappear as rapidly as they before decreased slowly.

This treatment of dropsy of the ovary is by no means a novel one. Many years ago Sir James Simpson suggested it, in an article on ovarian dropsy, in the "Library of Medicine." Subsequently he tried it, but so severe an inflammation was lighted up that the woman died. Since then he never used it.

A Dr. Franklin, of St. Louis, Mo., recently reported a case of ovarian tumor, of three years' growth, disappearing after treating with electrolysis. He used three gold needles, and a twenty cup battery. The patient was treated at one sitting. "Preparing the patient for the operation, adjusting the battery, and applying over the tumor, which was the size of a large cocoanut, a heavy steel ring, forcibly held downwards by an assistant, I plunged into the sac, one at a time, the three gold needles prepared for the purpose. Now, placing the positive electrode upon the back, and the negative in contact with the needles, the current was kept up for nine minutes through the sac. The patient only complained of pain and smarting in the back." Soon it (the battery) was again applied, and the current prolonged sixteen minutes. The skin around the needle showed evidence of cauterization. Some febrile action ensued, lasting forty or forty-eight hours. Patient walked about in a week, and in three weeks the tumor had so far disappeared that it seemed no larger than a pullet's egg. Subsequently she reported that she never felt better in her life.

### ELECTROLYSIS IN TREATMENT OF PALATO-NASAL POLYPI.

Dr. Paul Burns communicates, in a long article to the *Berlin Clinical Weekly*, July, 1872, his experience with that of other operators—eight cases in all—in the destruction of nasal and other polypi by electrolysis. One pole is held in the hand, and the other, by means of a wire, is applied to the tumor.—(*The Clinic.*)

### HOW DO THE SPERMATOZOA ENTER THE UTERUS?

An article, with the above heading, appears in the *St. Louis Medical and Surgical Journal*, for September, 1872, from the pen of Dr. Joseph R. Beck, of Fort Wayne, Ind., and is certainly quite readable. The gentleman has made *two* observations, and he is thereby enabled to wipe out Montgomery, Koelliker, Byford, Sims, Thomas, and all lesser luminaries, with their years of patient labor and study, in a masterly fashion. After quoting from the foregoing writers, he proceeds to promptly demolish them by stamping their ideas of the mode of entrance of the spermatozoa into the uterus as *puerile* and *absurd*. The independent motion of the spermatozoa, and the capability of progression while in parts congenial to such progression, as the vagina and cervix uteri, are totally insufficient to explain the point. The writer is certain that no mention has ever been made of the *true* mode of this entrance. He says:

"The only manner in which this subject can be positively set at rest, is to observe the os and cervix uteri *during the sexual orgasm. I have made two such observations, and know, beyond the peradventure of a doubt, that all the descriptions of the modes of entrance of the semen into the uterus, heretofore described, are totally and wholly, both in the main and detail, theoretically and practically untrue.*"

These two observations were made within twenty-four hours of each other. He copies from his case book:

"Aug. 7, 1872. Mrs. H. L., æt. 32 years, of strongly-marked nervous temperament—had one child—one abortion—last pregnancy six years ago," etc., etc. Diagnosis he determined was prolapsus uteri in second stage. Calling on his patient the ensuing day to adjust a McIntosh stem pessary, he was admonished to be very careful as he made the digital examination, as she was "very prone, by reason of her passionate nature, to have the sexual orgasm produced by very slight contact with the finger. Indeed, she stated, that this had more than once occurred to her when making digital examination of herself. Here then was an opportunity, never before offered to any one to my knowledge, and one not to be lost upon any consideration. Carefully separating the vulvæ with my left hand, so that the os uteri was brought clearly into view in a strong light, I swept the right forefinger across the cervix twice or three times, when almost immediately the orgasm occurred, and the following is what was presented to my view:

"The os and cervix uteri had been firm, and generally in a normal condition, with the os closed, so as not to admit the uterine probe without difficulty; but immediately the os opened, to the extent of fully an inch, made five or six successive gasps, drawing the external os into the cervix each time powerfully, and, at the same time, becoming quite soft to the touch. All these phenomena occurred within the space of twelve seconds time certainly, and in an instant all was as before; the os had closed, the cervix hardened, and the relation of the parts had become as before the orgasm."

He states that there was "intense congestion of the parts during the 'crisis.'" He questioned her "as to the nature of the sensations experienced by her at the period of excitement, and she is very positive that they were the same in *quality* as they ever were during coition, even before the occurrence of the prolapse, but admits that they were not exactly the same in *quantity*, believing that during coition the orgasm had *lasted longer*," etc. He likens the action of the os during orgasm to the mouth of a "sucker—a species of fish," that "passes the water through the mouth and out at the gills. * * * Precisely such a motion does the uterus make during the period of sexual excitement."

Of course, no conception takes place unless there be an opening of the os uteri during copulation, *i. e.*, unless there be orgasm present. Consequently, Drs. Montgomery, Koelliker, Byford, Sims, Thomas, and all other physicians, are to disbelieve the statements made to them by many of their patients, that they never experience sexual orgasm and yet bear children. Conceptions without penetration *must* be myths.

### CURE FOR THE ITCH.

The following prescription having been recommended for the cure of the itch by a distinguished dermatologist of Paris, and, as I have seen it employed with unfailing success, I take the liberty of transcribing it for the benefit of your readers:

R. Carbolic Acid, one drachm;
Water, one pint.

Or, what is still better, an ointment of—

Carbolic Acid, two drachms;
Benzoated Lard, four ounces.

Three or four frictions in the twenty-four hours suffice to kill the acari, after which a bath of soap and water is to be taken, and the disease produced by these parasites is thus infallibly cured in twenty-four hours.—(*Paris Correspondence of the London Medical Times and Gazette.*)

### THE USES OF THE UVULA.

Sir Duncan Gibb, Bart., M.D., LL.D., thus sums up his conclusions on the use of the uvula, in the *London Lancet:*

1st. It acts as a sentinel to the fauces in exciting the act of deglutition when anything has to be swallowed.

2nd. It compresses the soft palate, and holds its posterior free border firmly against the wall of the pharynx in deglutition, so that nothing can pass upwards.

3rd. It modifies speech in the production of loud declamation and the guttural forms of language, by lessening or diminishing the pharyngo-nasal passage when it acts as an elevator.

4th. Its elevating power is increased to the most extreme degree in the highest ranges of singing voice, and is very moderately exerted in the low ranges.

5th. Therefore, in its uses, deglutition and vocalization are the functions that are intimately associated with the uvula, and become impaired, more or less, if it is destroyed, wholly removed, or seriously injured.

### MUSCLE AS A CONSTITUENT OF NERVE TISSUE.

(*Gaz. Med. Ital.*, Nro. 52, 1871.) "Tigri has shown the presence of muscular fibres among the nerve-fibres of the cerebrospinal nerves, and the ganglia of the sympathetic. When an irritation is applied, contraction is caused, and the liquid contents of the nerve-fibres are caused to vibrate, and the motion is conveyed thus centripetally and centrifugally. In reference to these anatomical and physiological peculiarities, he calls the ganglia of the 'sympatheticus magnus,' as well as the large ganglia of the brain, 'nerve hearts.' He considers that the so-called Remak's nerve-fibres are muscular fibres."—(*N. Y. Medical Journal*, Sept., 1872.)

### NEW PLAN OF EXTRACTION OF BODIES FROM THE EAR.

Dr. Loewenberg, of Paris, describes a new plan for extracting solid bodies from the ear, as follows: A very small brush is made by rolling and fixing a narrow strip of old linen around a thin wooden handle (a match, for instance), and unraveling its free border to the length of a quarter of an inch. The end of the so-obtained fringe is dipped into a warm and very concentrated solution of glue, and applied to the visible part of the foreign body, or, rather, the operator leans it against the body by letting it glide very softly, and without exercising any pressure, over it. Previous to the application, the patient seats himself comfortably in an arm chair or on a sofa, and inclines his head towards the healthy ear. He remains in this posture from three-quarters of an hour to an hour after the introduction of the agglutinated brush. This time past, consolidation is generally accomplished and the foreign body can be extracted by gently pulling at the brush.—(*Medical Times and Gazette.*)

### ATOMIZED TURPENTINE WATER IN CHRONIC BRONCHITIS AND CONSUMPTION.

Dr. S. Goodwin, of Victoria, Texas, writes that he has lately been using turpentine water, inhaled by means of an atomizer, with signal advantage in cases of chronic bronchitis and consumption, where there was copious expectoration of either mucus or pus, with hemorrhage or violent cough. The turpentine water is prepared with spirits of turpentine by magnesia, in the same way that aromatic waters are commonly prepared by druggists.—(*Medical News.—New Remedies.*)

### SEASICKNESS.

Dr. Alderson, in a recent article, in the *British Medical Journal*, promulgates a new theory for this malady. He claims that *shock*, or *cerebral congestion*, is produced by the ship's motion, and from it result all the horrors of *nausea marina*. The sudden and enormous fall of the ship deranges the brain circulation, by suspending temporarily the force of gravitation in bringing the venous blood from the head, and thus is lessened the weight of blood that the heart has to overcome; the heart, freed from bearing this weight, is enabled to propel blood to the head with increased force. Thus we have venous blood from the cerebral vessels retarded and arterial blood accelerated, and there results general congestion of all the brain capillaries. This explanation is well elaborated by Dr. Macdonald, of the Harvard Medical School. It is premised that this effect is produced upon the human system while it is in the upright position. So far—good; but will the theory hold good when persons are lying down? Every one who has been seasick knows that lying down by no means obviates nausea and vomiting. Again, if this theory be true, why are not seamen continually suffering from cerebral congestion? Why are persons traveling not seasick after a few days' inuring to the ship's motion?

Chambers advances the theory, that seasickness depends on the relaxation of the œsophageal sphincter from temporary paralysis of the involuntary plexus which supply the fibres of that sphincter —said paralysis being produced by the motion of the ship.

Dr. Chapman, of London, says, "The proximate cause of seasickness consists in an undue amount of blood in the nerve centres."

### CANCER TREATED WITH CARBOLIC ACID.

The *American Journal of Medical Sciences* contains a report from Surgeon Bill, U. S. Army, of four cases illustrative of this treatment. Two of them were "cured," and the fourth was under treatment at the time of the report.

1st Case. An old man; had had labial epithelioma previously removed, and the disease returned in the cicatrix and could not be removed. Sixteen grains of acid daily for forty-nine days. Disease had then completely disappeared. That was three years ago, and no indications of a return are at present visible.

2nd Case. Similar in situation to the foregoing. Had been excised twice. After its third appearance, carbolic acid, fifteen grains daily, internally, was given. Disease disappeared, and no return noticed thereafter.

Dr. Bill thinks the acid ought to be diluted freely—three grains to the ounce of water.

### A NEW DIFFERENTIAL TEST FOR CARBOLIC ACID.

Surgeon Morson, of London, affirms that glycerine will completely dissolve carbolic acid, and will not dissolve creosote. A substitution of the one for the other, makes it very desirable that a ready means of distinguishing them be at hand.

### ASPIRATOR TROCAR.

M. Labbe, a young surgeon and sub-professor of Paris, has been doing wonders with the capillary or aspirator trocar. He has, in many cases, punctured the bladder above the pubis, and emptied it, where it was impossible to draw off the urine with a catheter. He prefers Dieulafoy's trocar, as being perfectly innocuous, the wound healing immediately. Many Parisian surgeons already predict one great disadvantage in this new method, not to patients, but to the future generation of surgeons, as catheterism would run the risk of being altogether put aside. At first, this instrument was only used as an explorer, and was not much larger than an ordinary urethra syringe. This was gradually increased in size, and was then employed for emptying large abscesses and cavities containing liquid. Still later it is so improved as to be adapted to washing out or injecting abscesses or cavities. It may yet even supersede the lancet for phlebotomy, as the risk of air entering is *nil*, and the ligature above the elbow can be dispensed with.

### REMOVAL OF PARIS PLASTER BANDAGE.

A strong solution of common salt causes the plaster to crumble, so that the bandage can easily be cut. It also quickly cleanses the hands of the operator from the gritty article used for securing immobility.

### STRANGULATED HERNIA REDUCED BY TAXIS AFTER PUNCTURING THE INTESTINE.

M. Demarquay advocated, before the Academie de Medicine, on May 21, 1872, the puncturing the gut with a small trocar in certain cases of hernia, and letting out the liquid and gas by means of Potain's aspirator. His operation on an hernial tumor, thirty-six hours old, after unsuccessful attempts at reduction by taxis and treating with ice, was a marked success. The patient's features had undergone great change, and fever was set up. A congenital, elongated, voluminous, inguinal hernia was found to exist. Having never succeeded in curing this description of hernia by operation, he resolved on puncture. About 120 grammes of intestinal liquid were drawn out, besides considerable gas. The tumor collapsed completely—trocar was withdrawn, and some minutes were allowed to elapse, to see if the tumor would again fill. No renewal of the tumefaction took place, and very slight pressure returned the tumor into the abdominal cavity. Quiet, low diet, and small doses of opium, completed the cure. No ill consequences.

M. Demarquay proposes to apply this new mode of treatment: 1. In all congenital hernias, and to recent hernias which become strangulated at the time of their formation. 2. To old hernias which were quite reducible a few days prior to strangulation, and in large umbilical hernias that have become recently strangulated. Aspiration should be employed at an early period, when one can be well nigh certain of returning into the abdomen the intestine in an unaltered state and capable of resuming its functions.

### THE MONOBROMIDE OF CAMPHOR.

Dr. W. A. Hammond has used this new preparation—consisting of one equivalent of camphor with one of bromine ($C._{10}$ $H._{16}$ O. Br.)—with marked success in infantile convulsions due to the irritation of teething. His experience in this difficulty is limited to two cases. "In each, a grain was given hourly, rubbed up with a little mucilage of acacia. Three doses were sufficient in one, and two in the other case. The children were aged respectively fifteen and eighteen months." In cases of "headache occurring in women and young girls, due to mental excitement and excessive study," it was employed with excellent effect. One dose, of four grains, generally sufficed.

In wakefulness, he thinks it "greatly inferior to the bromide of calcium, or even other bromides."

In a case of delirium tremens, Allen McLane Hamilton, M.D., (in *New York Medical Journal*, July, 1872,) asserts that the monobromide of camphor, in five-grain doses, was of excellent service. He also says that he is convinced that it is superior to any combination of camphor and opium in chordee.

### INSTANT ARREST OF EPISTAXIS.

Dr. Marin, of Geneva, states, in the *Jour. de Med. et de Chirurg. Practique*, May, 1872, that, as the bleeding in epistaxis generally flows from only one nostril, and most frequently from the anterior third of one of the nasal fossæ, he was led to believe, that by compressing the corresponding facial artery on the superior maxillary bone, near the ala of the nose, the afflux of blood would be diminished, and the hemorrhage at once be arrested. He has tried this plan in very many serious hemorrhages from the nose, and the expedient has proved perfectly and promptly successful.—(*L' Union Medicale*, 25th May, 1872.)

M. Gubler, of Hopital Beaujon, Paris, recommends glycerine, in tablespoonful doses, morning and evening, in cases of acne punctata—(*Paris Cor. London Times and Gazette.*)

### CURE FOR COLDS.

A Berlin correspondent of the *Chemist and Druggist*, recommends the following as a cure for this annoying affection:

"A wide-mouthed glass-stoppered bottle is filled with cotton and the latter is saturated with a mixture composed of—

| | |
|---|---|
| Acid Carbolic (pure), - - - - | scr. iv.; |
| Liq. Amm. (sp. gr. 0.960), - - - | oz. j. ss.; |
| Distilled Water, - - - - | oz. ij.; |
| Alcohol, - - - - - - | oz. iv. |

The vapors are drawn into the nose frequently during the day, and now and then inhaled into the mouth."—(*The Pharmacist.*)

### SUBCUTANEOUS INJECTIONS OF ERGOT IN UTERINE FIBROMA.

Professor Hildebrand, of Konigsberg, Prussia, draws attention to this subject in the *Berlin Clinical Weekly*, No. 25. In 1870 he first used it, daily, on a Polish woman, 33 years old, who had a large fibroma of the uterus, of three or four years' growth. Her hemorrhages were profuse, and nothing previously had succeeded in arresting them. To check them he resolved on ergotine injections, daily, till her next menstruation, when the injections were suspended temporarily. After the menstrual period, which was less painful and profuse than usual, the patient said the tumor was smaller. Measurement confirmed her assertion. The injections were resumed, and from week to week the tumor decreased, till, at the end of the fifteenth week, all vestige of it had disappeared. Other cases were tried, with the same effect—complete disappearance of the profuse hemorrhages, the debilitating serous discharges and the severe pains. Contraction of the nutrient vessels of the tumor doubtless results from this exhibition of ergot, and this strangulation of the growth is speedily followed by degeneration and absorption.

The solution used is composed of three parts of the watery extract of secale cornutum, to seven parts of distilled water and seven parts of glycerine. Langenbeck uses the *alcoholic* solution of ergot, but this is more painful, and more prone to give rise to suppuration, than the glycerine solution. The parts about the navel were chiefly used for injections, because the lower abdomen seemed more sensitive. A few days before and after menstruation slight hemorrhages follow the punctures, and after ten or fifteen daily injections, the solution flows out again from the orifices. To correct these points, wadding, wetted with collodion, was used immediately after each injection. About a drachm was used daily.

### THE CHARACTERISTICS OF SEX IN THE HUMAN SKULL—CONCLUSIONS OF MANTEGAZZA.

We have as yet no fixed characteristics to differentiate the male and female skulls. Very often the feminine cranium assumes the masculine type, and *vice versa.* The marked development of the superciliary arch is the most constant characteristic of the masculine skull, and, by it, we may determine the sex with a certainty almost absolute. The small size of the cranium, its diminished height, the less marked development of the points for muscular attachment on the occipital bone, are the distinctive characteristics most constant in the female skull; if, at the same time, the superciliary arch be almost completely absent, the female sex is almost certainly determined. This is all that science may, as yet, affirm in this regard, and that this may be established as a verity, it is necessary to examine these cranial differences in all the different races.—(*Archivis di Roma fascie*, 2, 1872.—*The Clinic.*)

### QUININE.

Dr. Greenhill (It. Monteverdi), of Cremona, Italy, has treated of the effect of this drug on the uterus, in a lately-published treatise, of which the *London Medical Times and Gazette* gives the following summary:

"Bark and its preparations act on the sympathetic, then on the spinal nerves. Thus it produces contraction of the muscular fibres, supplied by the great sympathetic, and especially of the womb, bladder, intestines, and blood vessels. Its effects depend on the dose, and on the condition of the organs acted on. It may restore relaxed organs to their normal state of tone; or, if the tone of these organs be already sufficient, it may induce morbid and excessive contraction. This is shown by its action on the pregnant womb, and especially during parturition. It may, administered imprudently, cause abortion; but in case of languid and feeble uterine contraction, it may accelerate childbirth, and cause the normal expulsion of the placenta. Dr. Monteverdi believes it to be far preferable to ergot, and less detrimental to mother and child. It takes the place of ergot in all relaxed conditions of the womb, menorrhagia, amenorrhœa and the like. It is the best preventive of puerperal fever, and the best remedy for its early stages. It is injurious in all cases of uterine excitation."

### WAR IN LEIPSIC.

"The battle rages hot and fierce in the German Congress of Physicians and Physicists now in session in Leipsic. It is a life-and-death struggle between Vienna and Berlin. The last number of the *Vienna Medical Press* (August 18), contains, in heavy type, the following special

*Telegram !*

"LEIPSIC, *Aug.* 15, '72.

"In the scientific encounter to-day, between Cohnheim and Stricker, before a brilliant audience, the four Professors chosen as referees, confirmed the statements of Stricker and established the correctness of his preparations. The attempt of Cohnheim to depreciate the scientific investigations undertaken in Vienna, and the Vienna method of work, was repudiated by a stormy protest of the whole association."—(*The Clinic.*)

### PERSONAL.

Dr. O. Liebreich, the discoverer of chloral's action, has been appointed Ordinary Professor of Materia Medica in the Berlin University.

Dr. Brown Sequard has resigned the chair of Comparative and Experimental Physiology in connection with the Faculty of Medicine in Paris, which he has held for several years. He is soon expected in New York.

Professor Virchow has recently been elected Dean of the Medical Faculty of Berlin University.

Professor T. Gaillard Thomas has resigned the chair of Obstetrics in the Medical College of Physicians and Surgeons of New York.

### PATHOLOGICAL ANATOMY OF DIPHTHERITIS.

The "*Gaz. Hebdom.*," August 2, 1872, contains a paper on the "Gangrenous or Diphtheritic Angina and Croup," by MM. Bouchut and Labadie-Lagrade, read before the Academy of Sciences. The following are their conclusions: In these diseases there are two kinds of anatomical lesions—the one, primary, due to the ulceration of the mucous membrane, or the presence of false membranes; and the other, secondary, cardiac or embolic. The former are well known, but the secondary—cardiac and pulmonary embolic lesions—have not as yet been described, and deserve to be known, explaining, as they do, the occurrence of death through an entirely special lesion of the lungs and other organs. In the heart there is almost always (fourteen times out of fifteen) an *endocardite vegetante*, with fibrinous deposits, which are the origin of frequent emboli. The lungs frequently (forty-five times out of one hundred and eighty,) contain knots of pulmonary apoplexy, or bloody infarcti, due to arterial emboli, such infarcti being sometimes colorless at the centre, and surrounded by a zone of hyperæmic pulmonary tissue. Sometimes there are knots of purulent infiltration, or even true metastatic abscesses. The lungs also frequently present at their surface, between the lobules, small venous thrombi. These bloody infarcti are sometimes found under the pericardium, among the altered muscular fibres of the heart, and in the subcutaneous cellular tissue, where small metastatic abscesses may be formed. Venous thrombi exist also in the pia mater, the brain, the liver, and in different parts of the body. With these lesions there always co-exists a more or less pronounced leucocytosis, which, in a bad case, is very considerable.—(*London Medical Times and Gazette*, Aug. 17, 1872.)

